# Red Fruits: Extraction of Antioxidants, Phenolic Content, and Radical Scavenging Determination: A Review

**DOI:** 10.3390/antiox6010007

**Published:** 2017-01-19

**Authors:** Gádor-Indra Hidalgo, María Pilar Almajano

**Affiliations:** Chemical Engineering Department, Universitat Politècnica de Catalunya, Avinguda Diagonal 647, Barcelona E-08028, Spain; chemicontact@gmail.com

**Keywords:** red fruit, berry, antioxidant extraction, scavenging assay, phenolic content

## Abstract

Red fruits, as rich antioxidant foods, have gained over recent years capital importance for consumers and manufacturers. The industrial extraction of the phenolic molecules from this source has been taking place with the conventional solvent extraction method. New non-conventional extraction methods have been devised as environmentally friendly alternatives to the former method, such as ultrasound, microwave, and pressure assisted extractions. The aim of this review is to compile the results of recent studies using different extraction methodologies, identify the red fruits with higher antioxidant activity, and give a global overview of the research trends regarding this topic. As the amount of data available is overwhelming, only results referring to berries are included, leaving aside other plant parts such as roots, stems, or even buds and flowers. Several researchers have drawn attention to the efficacy of non-conventional extraction methods, accomplishing similar or even better results using these new techniques. Some pilot-scale trials have been performed, corroborating the applicability of green alternative methods to the industrial scale. Blueberries (*Vaccinium corymbosum* L.) and bilberries (*Vaccinium myrtillus* L.) emerge as the berries with the highest antioxidant content and capacity. However, several new up and coming berries are gaining attention due to global availability and elevated anthocyanin content.

## 1. Introduction

In developed countries, alimentation is more focused on complimentary aspects than merely covering major component needs. Because of this, the so called red fruits, or berries, have recently attracted a lot of attention for their antioxidant properties, which are related to the high concentration of polyphenols present in them. In addition, their consumption worldwide has notoriously increased, and red fruits are nowadays not only consumed fresh but also used in cosmetics and dietary supplements.

To benefit from these molecules in nutraceuticals, creams and functional foods, an extraction needs to be performed in order to obtain an antioxidant-rich concentrate from a variety of edible berries.

The habitual aim is to obtain the maximum extraction yield of the compounds of interest, those that have more antioxidant activity, and, therefore, are capable of being more beneficial to human health, as well as being substitutes for synthetic preservatives, the latter having gained bad press over recent years, especially when part of the final product.

In the last few years, several studies analyzing the composition and the antioxidant properties of typical red fruits have been published frequently, and wide research has been taking place all over the world to find the optimal extraction methods to obtain richly antioxidant products for a range of berries. Although conventional solvent extraction is the most widespread technique for the extraction of antioxidant compounds from red fruits, new non-conventional methods have surfaced as environmentally friendly alternatives to the former method, such as ultrasound [1], microwave [2], and pressure assisted extractions [3], applied alone or together with solvent use, to reduce the energy and solvent requirement.

Although extraction techniques seem to have received much attention from researchers, the effects of cultivar [4], storage [5,6], and drying techniques [7,8] have also been studied. 

This review gathers some of the latest results published in scientific journals about antioxidant extraction and activity of red fruits, in order to facilitate a wider vision of this topic.

## 2. Phenolic Acids and Anthocyanidins in Red Fruits

Berries are characterized by the high amount of antioxidant molecules. These chemical compounds are a group of secondary metabolites that prevent the fruit from oxidation due to environmental factors, such as light, air, oxygen, and microbiological attacks. Phenolic antioxidants interfere with the oxidation process as free radical terminators and sometimes also as metal chelators.

Phenolic compounds or polyphenols are a group of hydroxylated molecules very susceptible to oxidation. Several studies have found them to have various biological properties, such as anti-proliferative, anti-diabetic, anticancer, anti-microbial, anti-inflammatory, antiviral, and especially important for this review: antioxidant [9]. They have different structures but in general contain an aromatic ring with one or more hydroxyl groups.

The radical scavenging capacity of phenolic antioxidant molecules is based on the ability to become radicals that are more stable compared to the majority of free radical species, due to the stabilization of the free electron by delocalization on the aromatic ring of the phenolic compounds.

A classification of phenolic antioxidants can be made, the most important being phenolic acids and anthocyanidins, as a subgroup of flavonoids.

**Figure 1:**
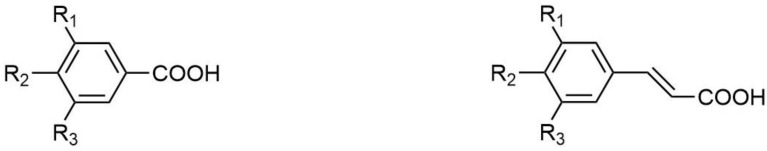
Chemical groups of each acid derivative.

**Figure 2:**
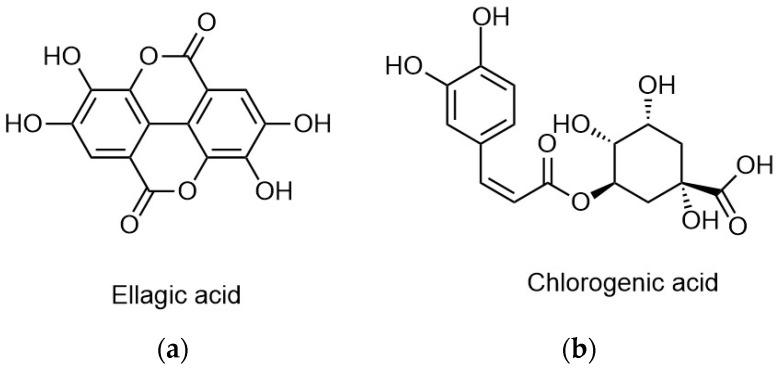
(**a**) Ellagic acid structure. (**b**) Chlorogenic acid structure.

Phenolic acids can be divided into two categories: hydroxybenzoic acid derivatives and hydroxycinnamic acid derivatives (Figure 1). The first group includes molecules such as hydroxybenzoic, gallic, vanillic, and ellagic acid (Figure 2a). In the second group p-coumaric, caffeic, ferulic, chlorogenic (Figure 2b), and hydroxycinnamic acid can be found.

These compounds can be widely found in berries, and each type of berry contains a characteristic profile of phenolic molecules.

**Figure 3:**
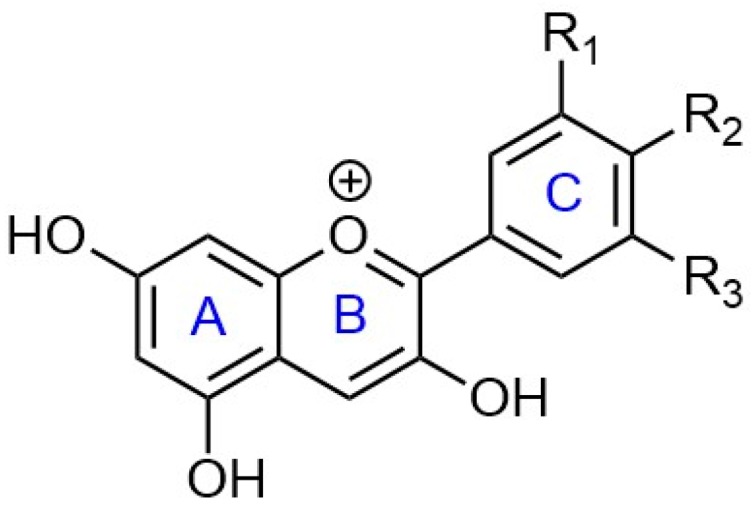
Flavylium ion structure and chemical groups of the anthocyanidins present in red fruits.

Anthocyanins are water-soluble plant pigments responsible for the blue, purple, and red color of many plant tissues [10]. Anthocyanidins are based on the flavylium ion, or 2-phenylchromenylium. The variety of chemical groups that can substitute the different positions (R1, R2…) create the anthocyanidins found in nature. A simplification of this ion, focusing on the common structures in red fruits can be seen in Figure 3.

There are about 17 anthocyanidins found in nature, whereas only six of them, cyanidin, delphinidin, petunidin, peonidin, pelargonidin, and malvidin, are present in most foods [11].

**Figure 4:**
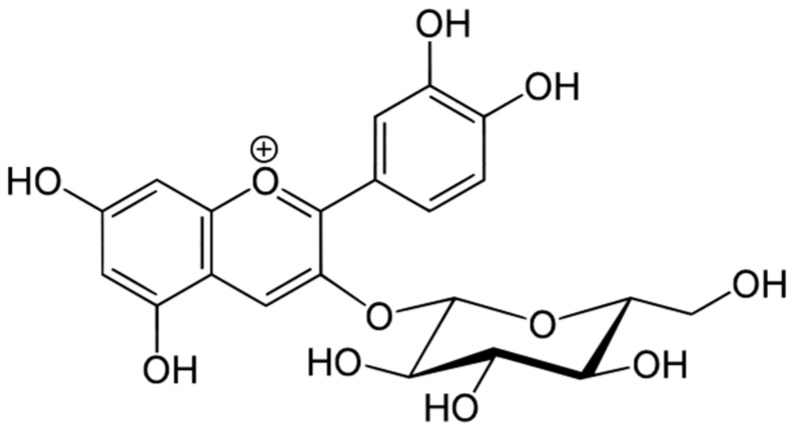
Cyanidin-3-glucoside.

When anthocyanidins are coupled to sugars, anthocyanins are formed. In red fruits, anthocyanins are mostly 3-glucosides of the anthocyanidins, cyanidin-3-glucoside being the most common compound in the majority of berries (Figure 4).

Among flavonoids, anthocyanins are antioxidants that play an important role in reducing the risks of various human degenerative diseases [3].

In general, the stability of anthocyanidins is pH-dependent. At acidic or basic pH the highly conjugated phenolic groups of the anthocyanidins protonate and deprotonate causing a change in electronic distribution which, at the same time, affect the absorption wavelength and the perceived color.

## 3. Berries and Red Fruits: General Characteristics and Antioxidant Compounds

The term “red fruit” or “berry” is used to name the small fruits, sweet or bitter, juicy and intensely colored (usually red, purple or blue) that grow in wild bushes, can be eaten whole, and lack objectionable seeds. The most well-known red fruits are strawberry, raspberry, blueberry, blackberry, and cranberry, which are also the ones with the most accessible information about them. Chokecherries, elderberries, mulberries, and other less frequent fruits are also commonly considered as red fruits.

Berries, in general, are rich in sugars (glucose, fructose), but low in calories. They contain only small amounts of fat, but a high content of dietary fiber (cellulose, hemicellulose, pectin); organic acids, such as citric acid, malic acid, tartaric, oxalic, and fumaric acid; and certain minerals in trace amounts [12].

In Table 1 there is a summary of the nutritional values for the most well-known red fruits.

In this section only the most typical berries will be commented upon. Later on in this review, several research papers will be referenced which not only use common but also novel red fruits for antioxidant extraction, due to the continuous appearance of berries from different parts of the world with interesting properties.

### 3.1. Fragaria spp.

*Fragaria* is a genus of flowering plant in the rose family, Rosaceae, commonly known as strawberry for their edible fruits. There are more than 20 described species and many hybrids and cultivars. The most common strawberries grown commercially are cultivars of the hybrid known as *Fragaria* × *ananassa*, which has a bigger fruit (around 3 cm wide and 4 cm long).

In a study conducted on strawberries using liquid chromatography for the identification of antioxidant compounds, four anthocyanins were readily found: cyanidin-3-glucoside, pelargonidin-3-glucoside, and possibly pelargonidin-3-rutinoside [13]. Identity of the first two anthocyanins was confirmed by spiking with authentic standards whereas pelargonidin-3-rutinoside was tentatively assigned by comparison of the peak online spectrum with a spectrum presented by another author.

Gallic acid (566 mg/kg) and syringic acid (0.12 mg/kg) were found in red strawberries [14]. Strawberries are an excellent source of potassium, fiber, many B vitamins, vitamin C, vitamin K, manganese, iodine, folate, omega-3 fatty acids, magnesium, and copper [15].

### 3.2. Rubus idaeus

*Rubus idaeus* (raspberry, also called red raspberry or occasionally as European raspberry to distinguish it from other raspberries) is a red-fruited species of *Rubus* native to Europe and northern Asia and commonly cultivated in other temperate regions.

Raspberries have very interesting nutritional properties due to their high amount of fiber and antioxidant compounds, including phenolic acids, flavonoids, and lignans with a reduced calorie input. The presence of ellagitannins and anthocyanidins not only contribute to the healthy attributes but also to their attractive color [16]. Quercetin is the most representative flavonol in red raspberries [17].

### 3.3. Rubus fruticosus

Blackberry (not to be confused with black raspberry) is a bushy plant in the rose family, native to Europe, Asia, and North Africa. It was found that cyanidin-3-glucoside (representing 92.76% of total anthocyanins) was the major anthocyanin in blackberry extract [18]. This result is in agreement with data reported by other authors [11]. Flavanols were also found, specifically (−)Epicachetin was found in 120–620 mg/kg FW (fresh weight) by micellar electrokinetic chromatography [19].

### 3.4. Vaccinium corymbosum

*Vaccinium corymbosum*, the northern highbush blueberry, is a North American species of blueberry which has become a food crop of significant economic importance. Recent studies proving the effectiveness of blueberries as a good source of antioxidants, necessary for a balanced diet and the added anticancer properties have resulted in this fruit achieving more popularity around the world. The increasing demand is being covered with higher production, especially from the American continent, which delivers more than three quarters of the global production of this fruit.

### 3.5. Vaccinium myrtillus

Three times smaller than the blueberry (*Vaccinium corymbosum*), but similar in appearance and flavor, *Vaccinium myrtillus* is also known as the European blueberry, or bilberry. Several clinical trials demonstrated the benefits of *Vaccinium myrtillus*-extracted anthocyanosides in the management of visual disorders in humans [20]. The main anthocyanins found in bilberry extract are cyanidin-3-glucoside (14.33%) and delphinidin-3-glucoside (13.45%), followed by malvidin-3-glucoside (11.18%), petunidin-3-glucoside (10.73%), and delphinidin-3-galactoside (8.98%) [18].

### 3.6. Vaccinium macrocarpon (America)/Vaccinium oxycoccos (Europe)

Cranberry is a wild, evergreen dwarf shrub of the Ericaceae family which grows in marshy coniferous forests and bogs. Common cranberry (*Vaccinium oxycoccos*) and the similar looking small cranberry (*Oxycoccos microcarpus*) are evergreen dwarf shrubs with small, narrow leaves and red edible fruit. American cranberry (*Vaccinium macrocarpon*) is a major commercial crop in eastern Canada and north-eastern USA [21].

Quercetin is one of the major significant flavonoids occurring in cranberries. Ellagic acid represents 51% of the total phenolic compounds in the berries, and cyanidin-3-glucoside is the dominant anthocyanin [12].

While the previously described berries are widely known, there are many others which have been studied for their antioxidant capacity, whether they can be found worldwide or only grow in restricted areas, which are mainly studied or consumed by the local population.

## 4. Most Common Antioxidant Content Determination and Radical Scavenging Assays in Red Fruits

In this section the key information about antioxidant content is highlighted, and the most commonly used assays to determine the quantity of these molecules and their antioxidant power are explained.

### 4.1. Antioxidant Content Determination

The determination of antioxidant content can be done either by chromatography methods, such as High Performance Liquid Chromatography (HPLC) coupled with a Diode-Array-Detector (DAD), Mass Spectroscopy (MS) or fluorescence detector; or using other less specific colorimetric methods.

#### 4.1.1. TPC

Total Polyphenol Content, (TPC) can be determined by colorimetric spectrophotometry with the Folin-Ciocalteu reagent method. This reagent contains complexes of fosfomolibdic/fosfotungstic acid [22]. The chemical reaction is based on the electron transfer from phenolic compounds and the measurement of the absorbance of the blue colored complexes at 725 nm. Gallic acid is used as a standard, and results are usually expressed as mass of Gallic Acid Equivalents (GAE) per gram of mass of the sample or extract.

One of the main disadvantages is that the TPC method is not specific, as the reagent can be reduced by other compounds other than phenolics [23]. The high values obtained for TPC could result from interference of other reducing substances, such as ascorbic acid or reducing sugars [22].

#### 4.1.2. TAC

Total Anthocyanin Content (TAC) is usually found with the pH-differential method. Anthocyanin pigments undergo reversible structural transformations with a change in pH manifested by different absorbance spectra. The oxonium form predominates at pH 1.0 while the hemiketal (colorless) form at pH 4.5. The pH-differential method is based on this reaction and allows accurate and rapid measurements of the total amount of anthocyanins, even in the presence of polymerized degraded pigment and other interfering compounds [24].

Cyanidin-3-glucoside is used as a standard, and results are usually expressed as mass of cyanidin-3-glucoside (C3G) per gram of mass of the sample or extract.

#### 4.1.3. Ascorbic Acid Content

Also known as vitamin C, ascorbic acid and its derivatives are known to have antioxidant properties, acting both directly, by reaction with aqueous peroxyl radicals, and indirectly, by restoring the antioxidant properties of fat-soluble vitamin E [25].

Regarding red fruits, however, it has been found that polyphenols and anthocyanins contribute substantially to the antioxidant intake [26], while ascorbic acid only makes a minor contribution to the total antioxidant capacity [27].

Ascorbic acid content can be determined by a variety of methods, including titration, spectrophotometry, chromatography or voltammetry [28].

Results are usually expressed as mg ascorbic acid/100 g fresh weight [26].

### 4.2. Radical Scavenging Assays

Antioxidant capacity assays can be divided into two categories according to their reaction mechanisms: Hydrogen Atom Transfer (HAT) based assays and Single Electron Transfer (SET) based assays [29].

The first one, mainly found in non-ionizing solvents, consists in the transfer of a hydrogen atom from the substance that acts as the antioxidant to the free-radical. The HAT-based methods are generally composed of a synthetic free radical generator, an oxidizable molecular probe, and an antioxidant [30]. ORAC (Oxygen Radical Absorbance Capacity) assay is included in this category.

The second category, Single Electron Transfer (SET), detects the ability of a potential antioxidant to transfer one electron to reduce any compound, including metals, carbonyls, and radicals. It involves one redox reaction with the oxidant (also as a probe for monitoring the reaction) as an indicator of the reaction endpoint. Some assays included in this category are ABTS (2,2′-azinobis-(3-ethilenebenzotiazolin)-6-sulfonic acid), FRAP (Ferric Ion Reducing Antioxidant Power) and TPC, when using Folin-Ciocalteu reagent.

SET and HAT reactions may occur together and the mechanism finally dominating in a system is determined by the antioxidant characteristics. DPPH (2,2-diphenyl-1-picrylhydrazyl radical) assay can be included in both categories.

Each radical scavenging assay relies on a colorimetric or fluorescent change due to the scavenging of the radicals added to the solution. In DPPH, ABTS, and FRAP methods, a colorimetric change is measured by spectrophotometry at a certain wavelength. In ORAC, antioxidant compounds in the sample inhibit fluorescence decay caused by the reaction of fluorescein with peroxyl radicals.

Results from radical scavenging assays can be expressed in different units, but usually DPPH is expressed either as inhibition %, representing the % of scavenged radicals from the total available. Another common way is the IC_50_, the concentration of antioxidant substance necessary to scavenge 50% of the free radicals. In this last case, the lower the IC_50_ value, the higher the antioxidant capacity obtained.

Both ABTS and ORAC are usually expressed as μmol (or mmol) of Trolox equivalents (TE) per liter. Trolox is a water soluble vitamin E analogue used as a standard scavenger.

FRAP assay results are expressed as mol Fe^2+^ equivalents, as this assay is based on the ability to reduce a yellow ferric complex (containing Fe^3+^) to a blue ferrous complex (containing Fe^2+^) by electron-donating antioxidants in an acidic medium.

**Figure 5:**
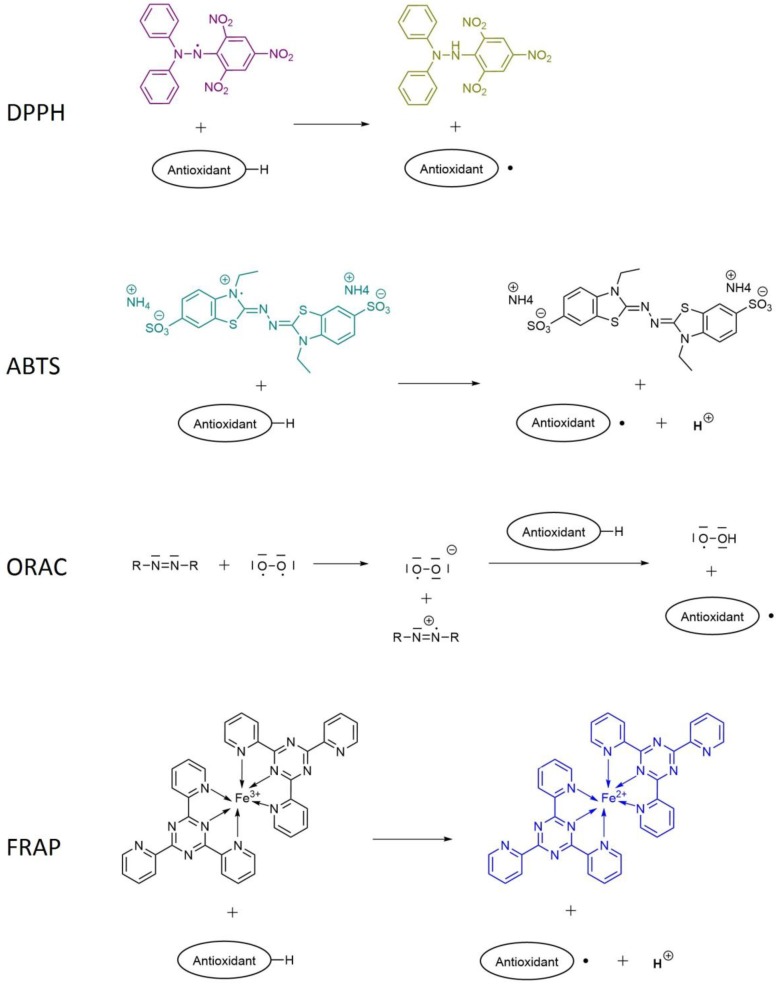
Chemical reaction involved in the most used scavenging assays [30,31,32,33,34,35].

The chemical reactions between the antioxidant sample and the reagent that take place in radical scavenging assays are summarized in Figure 5, and an overview of each assay can be seen in Table 2.

An important factor to take into account is the concentration of the extract with which the scavenging assay is made. Extracts with higher concentration lead to better results, higher antioxidant capacity samples. To undertake a correct comparison, this concentration should be taken into consideration when looking at different data.

## 5. Common Methods for the Extraction of Antioxidants from Red Fruits

The characteristics of the extract obtained from red fruits are determined by two main factors: pre-extraction factors and extraction factors. The first one determines the amount of antioxidants in the berries, while the second governs the ability to extract those molecules from the vegetable matrix.

The cultivars, season of harvesting, and geographic location of berries are important parameters that affect antioxidant content and activity of the final extracts. Climate, sunlight exposure, water intake from plants, and ripening stage when berries are collected are very difficult to control. This is why the majority of researchers focus on the optimization of extraction techniques from different berries.

The addition of some substances, such as BTH (benzo-thiadiazole-7-carbothioic acid S-methylester) [37], or the radiation of the berries with different light treatments, like ultraviolet [38] or blue light [39] could enhance the amount of antioxidant compounds and antioxidant capacity.

To perform the extraction, there are three elements involved: the red fruit, the extraction method, which can be classified into the chemical or physical assistance category (or both), and the influencing factors, such as time and temperature.

Regarding extraction methods, conventional solvent extraction is the most widespread technique for the extraction of antioxidant compounds from red fruits, especially at an industrial scale. But this method consumes a great amount of energy, due to the heating process and solvents necessary to achieve the solid-liquid extraction.

**Figure 6:**
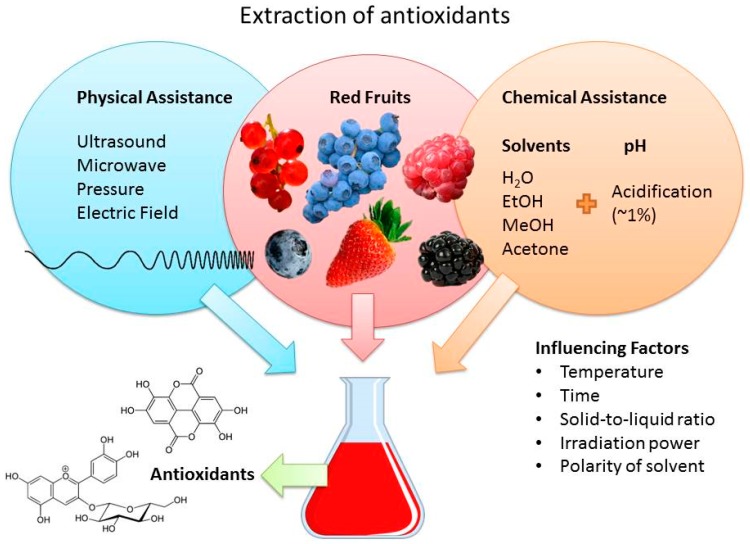
Scheme of the extraction of antioxidants from red fruits.

New non-conventional methods have emerged as environmentally friendly alternatives to the former method, such as ultrasound, microwave, and pressure assisted extractions, applied alone or together with solvent use, to reduce the energy and solvent requirement (Figure 6).

A statistical method known as Response Surface Methodology (RSM), based on a second order polynomial model is commonly applied to determine the best combination of process parameters to ensure maximal extraction efficiencies. According to the fitted polynomial model, a response surface plot is generated to determine the optimal conditions and maximal extraction yields. Compared to other statistical methods such as orthogonal design method and single factor experiment method, RSM can reduce the number of experimental trials and determine the interactive effects of process variables [40].

In the upcoming sections, a review of the main extraction methods will be discussed. While the new extraction techniques present serious advantages to the conventional methods, they also hold some disadvantages that should be considered when choosing an alternative (Table 3).

### 5.1. Physical Extraction

Cold press extraction is one of the most antique extraction methods. It allows extraction of antioxidant-rich inner fruit liquids, without need of heat or solvent addition. It is widely used nowadays for the production of fruit juices and oil extraction.

It is also used as a first step in the recovery of antioxidant compounds from red fruits, where a screw press is used to obtain a first liquid and successive extractions of the press residue increase total extraction yield [45].

### 5.2. Solvent Extraction

Traditionally, there are two main types, maceration and solvent extraction.

Maceration is the extraction of substances from a matrix by the release of them into a solvent, without heat application, over long periods of time. It is used by some researchers to obtain extracts rich in antioxidants [46].

Solvent extraction (SE) works with the same mass transfer phenomena, but heat application and the use of a variety of solvents allows extraction of target components in a shorter time. Stirring is commonly used as a mass transfer enhancing agent. Soxhlet extraction is widely used at laboratory scale, as it is inexpensive and does not need a subsequent separation by filtration [41].

Both extractions are usually performed using solvents such as water, ethanol, methanol, and acetone, both as monocomponents or mixtures. The solvent can be acidulated to enhance extraction, usually at a 1% amount, by using HCl, acetic acid or other acids.

Water-alcohol mixtures are more efficient than the corresponding mono-component solvent systems in extracting phenolic compounds. Specifically, varying ratios water–ethanol were tested and the extraction yields of polyphenols obtained with 50% ethanol (vol.) at different temperatures (20, 40, and 60 °C) were about 2-times higher than the yields of extraction using pure water.

Numerous studies have tested the effectiveness of different solvents for the extraction and recovery of antioxidant compounds, and ethanol has been shown to be the best when comparing it with water, acetone, hexane, ethyl acetate, and methanol [1,34].

It has been reported that the optimal composition is around 40%–70% ethanol in the water-EtOH mixtures [1,24,47,48,49,50,51]. Methanol mixtures, sometimes acidulated, are the second most used solvent [13,18,52].

Also, ethanol-water mixtures are one of the most used solvents due to the economic affordability and because ethanol can be obtained from a renewable source (sugar cane) and is classified as a GRAS (Generally Recognized As Safe) solvent, enhancing the green chemistry approach [50].

Several authors have reported differences in DPPH values depending on the type of solvent used for the extraction, as well as other factors such as the fruit drying method [3].

#### 5.2.1. Effect of Solid-to-Solvent Ratio

Solid-to-solvent ratio can be expressed as a ratio, such as 1:2, or as the product of the ratio, being 0.5. As the solid-to-solvent ratio is increased, less solvent will be used to extract the sample. This leads to a higher concentration of antioxidant compounds in the extract obtained. However, if there is a lack of solvent, the mass transfer is hindered. Therefore, there must be a balance between both elements.

#### 5.2.2. Effect of Temperature and Time

These two factors work cooperatively: increasing temperature leads to a need of less extraction time to obtain the same amount of antioxidants, while increasing time results in a lower temperature required.

An increase of temperature from 25 °C to 40 °C led to the increase of extraction yield up to 20% after the same extraction time (15 min) [51]. This had a positive effect on the radical scavenging activity of the extracts against the DPPH radical. The authors suggest this is due to an increase of the total anthocyanin content (cyanidin) in the extracts when increasing extraction time and temperature. This study showed that temperature increase in the given range had a more pronounced effect on the extraction yield than time.

A different author [1] found that at 60 °C the yields of extracted polyphenols were tripled compared to the yields obtained at 20 °C. The observed positive effect of temperature could be explained by the higher solubility of polyphenols in the solvent, the higher diffusivities of the extracted molecules, and the improved mass transfer at higher temperatures. However, a more recent study by the same author proved that at high temperatures (70 °C), a decrease of anthocyanins yield with time was observed, suggesting their thermal degradation at such conditions [10]. This idea is supported by another study [16] which claims that high temperatures coupled with exposure to molecular oxygen may degrade certain groups of bioactive compounds including anthocyanins, which are important antioxidants in red fruits.

### 5.3. Ultrasound Assisted Extraction

Ultrasound assisted extraction (UAE) is a non-thermal technique, which uses frequencies equal or above 20 kHz. Ultrasound is widely used in the areas of science and engineering because it is a non-thermal technique with multiple capabilities suitable for different industrial applications, including the food industry [16]. This method has gained particular attention due to low cost equipment, simplicity, and a higher efficiency compared to solvent extraction, because of reduced heat and solvent expense [42,51]. This positions it as a more environmentally friendly extraction method. The mechanism is as follows: ultrasound induces cavitation, which causes cell wall disruption. This allows permeation of intracellular compounds and therefore liberation of antioxidants and other molecules [44].

As anthocyanins are vacuolar pigments, which accumulate in the plant cell central vacuole, cavitation and cell disruption caused by ultrasound waves may enhance the mass transfer from the solid matrix to the solvent improving the extraction of anthocyanins [50].

In general, antioxidant compounds are found in higher concentration in the outer skin and seeds.

#### Effect of Sonication

Some authors [10] proved that ultrasound assistance improves considerably both yields of extraction of phenolics. They found out low frequencies (20 kHz) were enough to extract efficiently anthocyanins from *Aronia melanocarpa* and higher frequencies could cause degradation of these compounds.

Furthermore, others [16] extracted *Rubus idaeus* puree without any added solvent. Results showed that there was a significant drop in antioxidant activity after 30 min of sonication at the highest tested frequency (986 kHz). The observed drop was probably due to the synergic effect of the ultrasound and temperature increase due to the high frequency applied.

Recently, a group of researchers [17] studied the recovery of antioxidant compounds and their antioxidant activity from red raspberry and blueberry puree (*Rubus strigosus* var. Meeker and *Vaccinium corymbosum*). Contrary to what the majority of research found out, in their study, ultrasound had a deleterious effect, reducing the content of anthocyanins by 33%. Also, a decrease of 30% ascorbic acid was induced by UAE.

The same authors tested the rheological properties of these two purees before and after extraction treatments. The reduction in particle size due to sonication treatments is reflected in the lower apparent viscosity. Also, the behavior changed, especially in blueberry, shifting from non-Newtonian to an almost Newtonian model.

### 5.4. Microwave Assisted Extraction

Another non-conventional technique is Microwave Assisted Extraction (MAE). It is characterized by the generally low or lack of added solvent. The intrinsic moisture of fruit is used and, therefore, mass and heat transfer phenomena take place in the same direction, from the berry matrix to the liquid medium. Cells are damaged and intracellular content is released to the medium [2].

MAE’s greatest advantage is the fast heating, with a reduced equipment size [42]. This reduces both the extraction time and amount of solvent needed, which automatically causes a lower CO_2_ emission to the atmosphere [53].

Some authors designed an optimal solid-to-solvent ratio of 30%–34%. Higher or lower ratio cause poor extraction [54].

Microwave Hydrodiffusion and Gravity (MHG) is a type of MAE whose main characteristic is the fact that the extract glides through a perforated support and is gathered by gravity in a flask. In a study [2] using this technique, MHG extracts had much higher TPC and IC_50_ DPPH values than SE extracts.

### 5.5. Pressure Assisted Extraction

Pressure assisted extractions are green alternative methods that can be classified into pressurized liquid extraction and supercritical CO_2_ extraction.

#### 5.5.1. Pressurized Liquid Extraction

Pressurized Liquid Extraction (PLE) combines the conventional solvent extraction liquid with a pressure application. This allows the operation with high temperatures while the solvent remains liquid, enhancing solubility and kinetics. Thanks to this, extra extraction efficiency is achieved as it usually requires less time (5–30 min) and less solvent than SE [3].

#### 5.5.2. Sub/Supercritical CO_2_ Extraction

Supercritical CO_2_ extraction (SFE-CO_2_) is a non-conventional extraction technique that operates at very high temperature and pressure (supercritical conditions). This enables high mass transfer rates, difficult to achieve with liquid solvents. Therefore, the extraction time required is smaller [43].

As carbon dioxide is a very non-polar molecule, a polar solvent is needed to increase solubility. This added solvent (usually at 5%) is called “entrainer”.

Some discoveries made in several studies regarding this extraction method are that solvent density affects extraction, pressures should be higher than 220 bar [55], and the extracts are more active against ABTS than DPPH due to the steric impediment of the latter molecule [56].

### 5.6. Pulsed Electric Fields

In Pulsed Electric Fields (PEF), the sample is placed between two electrodes, and an electric field is applied in a pulsed way. Pulse amplitude ranges from 100 V/cm to 80 kV/cm and extraction times of less than a second, in repetitive cycles. This electric field causes damage on plant cell walls, which is known as ‘electroporation’. The formed pores allow the release of intracellular compounds into the liquid [42].

Table 4 summarizes the main results from a selection of research papers, classified according to the extraction method used. As the amount of data available is overwhelming, only results referring to berries were included, leaving aside other plant parts such as leaves [57,58], roots [59], stems [60], or even buds [61] and flowers [62]. Regarding time limit, articles from 2000 until June 2016 were considered, with a few exceptions.

The table includes the extraction conditions and efficiency, the antioxidant content and results of radical scavenging assays performed on red fruits.

The extraction conditions are schematized as follows: Solvent (volume%, the rest up to 100% is water unless indicated), solid-to-liquid ratio, temperature, extraction time. The complete botanical name of the red fruits named through this review followed by the common name can be found in Appendix A. A key for the abbreviations used can be found in Appendix B.

Due to the amount of information given in the previous table, a selection of results using different extractions methodologies applied to *Vaccinium myrtillus*, the red fruit with the highest antioxidant content and capacity is included in Table 5.

## 6. Applications

There are already applications obtained from studies regarding storage conditions. In a study [39] it was found that blue light during storage could enhance antioxidant content and capacity. There are commercially available fridges with blue LED lights constantly irradiating the fruit and vegetables compartment, and consumers have reported a longer food life when this technology is used.

With the boost of red fruit popularity, brands are using berry extracts as part of their ingredient list. The valorization of vegetable by-products facilitates the access of production plants to cheap raw ingredients, such as fleshy residues containing seeds and peel, rich in antioxidants, as a result of juice production.

The diminishment of synthetic preservatives in cosmetics and food products is a top priority for manufacturing companies, who care for the negative impact these artificial molecules have on their product’s label as well as to comply with the strict legislation on the use of synthetic food additives [101]. The use of red fruit extracts is being used in many cosmetic applications, while benefiting from it in several ways; it favors the Clean Label approach and it is a merchandising tool, as antioxidant properties of berries are seen as being transferred to the product.

However, there is still major work to do. Fruit extracts cannot always replace traditional preservatives, as antioxidant power and stability are more sensitive. Some pilot-scale extractions and simulations of real processes have been performed [45,99,102], but further studies are needed to assess the potential application of each red fruit, as new berries with interesting properties are continuously appearing. Although there are some in vivo studies [103,104], and reviews [35,105], it is necessary to evaluate the obtained extracts in specific products such as creams, nutraceuticals or functional foods.

## 7. Conclusions

Conventional solvent extraction is the most widespread technique for the extraction of antioxidant compounds from red fruits at industrial scale, but this method consumes a great amount of resources. New non-conventional methods have surfaced as environmentally friendly alternatives to the former method, such as ultrasound, microwave, and pressure assisted extractions, applied alone or together with solvent use, to reduce the energy and solvent requirement.

Although there is wide research on this topic, high variability concerning the results is still a major impediment to achieving global consensus. The spectrum of solvents, temperatures, irradiation power or pressure, and extraction time are influencing parameters on extraction yields and the activity of antioxidant extracts. Factors previous to extraction, such as cultivar characteristics, harvesting time, storage, and drying method also affect the phenolic content.

However, several researchers have drawn attention to the efficacy of non-conventional extraction methods, accomplishing similar or even better results using these new techniques. Some pilot-scale trials have been performed, corroborating the applicability of green alternative methods to the industrial environment. Unfortunately, high investment is still needed, which lowers the attractiveness due to the economic cost.

Among the most studied red fruits, blueberries (*Vaccinium corymbosum*) and bilberries (*Vaccinium myrtillus*) emerge as the berries with the highest antioxidant content and capacity, assets clearly correlated. Despite the leadership of the classic red fruits in production and use as nutritional supplements, several new up and coming berries are gaining attention due to their global availability and elevated anthocyanin content.

In vitro assays, especially radical scavenging, are an extended way to demonstrate antioxidant activity. Nonetheless, the results are not always applicable to in vivo situations. For this reason, further in vivo assays are necessary to prove real antioxidant capacity in cosmetic, food, and nutraceuticals.

## Figures and Tables

**Table:** 

Hydroxybenzoic Acid Derivatives	R1	R2	R3	Hydroxycinnamic Acid Derivatives	R1	R2	R3
*p*-Hydroxybenzoic	H	OH	H	*p*-Coumaric	H	OH	H
Protocatechuic	OH	OH	H	Caffeic	OH	OH	H
Vanillic	OCH_3_	OH	H	Ferulic	OCH_3_	OH	H
Syringic	OCH_3_	OH	OCH_3_	Sinapic	OCH_3_	OH	OCH_3_
Gallic	OH	OH	H				

**Table:** 

Anthocyanidin	R1	R2	R3
Pelargonidin (Pg)	H	OH	H
Cyanidin (Cy)	OH	OH	H
Delphinidin (Dp)	OH	OH	OH
Peonidin (Pn)	OCH_3_	OH	H
Petunidin (Pt)	OCH_3_	OH	OH
Malvidin (Mv)	OCH_3_	OH	OCH_3_

**Table 1:** Nutritional composition of common red fruits. From USDA nutritional database.

Tipical Values for 100 g	Energy (kJ)	Carbohydrate (g)	Fat (g)	Protein (g)	Vitamin C (mg)
Strawberry (*Fragaria x ananassa*)	136	7.68	0.3	0.67	58.8
Raspberry (*Rubus idaeus*)	196	11.94	0.65	1.20	26.2
Blueberry (*Vaccinium corymbosum*)	240	14.49	0.33	0.74	9.7
Blackberry (*Rubus fruticosus)*	180	9.61	0.49	1.39	21.0
Cranberry (*Vaccinium macrocarpon*)	190	12.20	0.13	0.39	13.3

**Table 2:** Advantages and disadvantages of the most used scavenging assays and their typical units [22,31,33,36].

Assay	Pros	Cons	Results Expressed as
DPPH	Simple, quick ^1^, inexpensive	Only organic solvents (lipophilic antioxidants), narrow pH range.	Inhibition %, IC_50_, mg AAE/L, mg GAE/L
ABTS	Very fast ^2^, wide pH range, hydrophilic and lipophilic molecules allowed	Long reaction time (>6 min) could give incorrect results due to short assay	Mol Trolox equivalents/L
ORAC	Involves variation of value with time, radical behavior similar to authentic radicals	High variability in results	Mol Trolox Equivalents/L
FRAP	Iron-containing food oxidation studies can benefit from this assay	Not all Fe^3+^ reductants are antioxidants, and some antioxidants are not able to reduce Fe^3+^	Mol Fe^2+^ equivalents

^1^ 20–60 min; ^2^ 6 min. AAE: Ascorbic Acid Equivalents.

**Table 3:** Qualitative comparison of extraction methods [3,16,41,42,43,44].

Extraction Method	Pros	Cons
Maceration ^a^	No additional energy needed	Very long extraction times.
Solvent extraction ^a^	Easy industrial scale-up. Well known technique	Long extraction times. Some solvents not valid for food/cosmetic industry
Ultrasound assisted	Higher efficiency (less extraction time and solvent consumption requirements ^b^). Safe extraction of heat labile compounds	Expensive scale-up
Microwave assisted	Quicker heating. Reduced equipment size. No added solvent needed	Risk of burning the sample and denaturalizing compounds
Pressure assisted	SFE-CO_2_ extraction: CO_2_ no toxicity, extraction in absence of air and light, very pure extracts	Expensive scale-up
Pulsed electric fields	Already acquired by some food industries to scale-up processes	Need of very specialized equipment

^a^ Conventional extraction methods; ^b^ Compared to conventional extraction methods. SFE-CO_2_: Supercritical carbon dioxide extraction.

**Table 4:** Extraction conditions and results obtained from a selection of studies.

Red Fruit	Extraction Conditions and Efficiency	Antioxidant Content	Radical Scavenging Assays	References
SOLVENT EXTRACTION				
*Hippophae rhamnoides*	MeOH (80%), 1:10, 5 min Yield: 17.6% DW	TPC: 741.9 mg GAE/g DW	DPPH: 5.36 mmol GAE/L	[2]
*Euterpe oleracea*	EtOH (70%–80%, acidified 0.065–0.074 M HCl), 1:4, 58 °C, 4 h	TPC: 432.13 mg GAE/100 g FW. TAC: 239.14 mg/100 g FW	ORAC: 6.87 mmol TE/100 g FW	[63]
*Aronia melanocarpa*	EtOH (80%), 1:25, 85 °C, 2 h	TPC: 919.7 mg of GAE/g DW. TAC: 1146–3715 mg C3G/100 g		[8]
*Ribes nigrum*	EtOH (60%), 1:100, 20 °C, 60 h	TPC: 37.85 mg CA/g FS DW. TAC: 13.59 mg C3G/g FS DW		[64]
*Rosaceae Fragaria, Vaccinium corymbosum, Rubus idaeus, Rubus fruticosus* and *Euterpe oleracea*	EtOH (80%), 15 min		DPPH (IC_50_ mg/mL): 0.70, 0.80, 1.40, 5.60 and >10 for *Vaccinium corymbosum, Rubus idaeus, Rubus fruticosus, Rosaceae Fragaria* and *Euterpe oleracea*, respectively	[65]
*Vaccinium myrtillus*	Water, 1:3, 80–100 °C, 4–15 min. Yield: 40%–68%	TPC: 576 mg GAE/100 g FW (1153 mg GAE/L extract). TAC: 332 mg C3G/100 g FW (625 mg CGE/L extract)		[66]
*Hippophae rhamnoides*	Water, 4:5, r.t., 10 min		DPPH: 71%	[67]
*Smilax aspera*	MeOH (acidified 0.1% HCl), r.t., 20 h	TAC: 23.7 mg CGE/g skin		[68]
*Dovyalis hebecarpa*	Acetone (20%, acidified 0.35% formic acid), 1:120, 17.6 min	TPC: 1421 mg GAE/100 g pulp FW. TAC: 319 mg C3G/100 g pulp FW		[69]
*Lycium barbarum*	MeOH (80%), 1:5, ovn.		DPPH: 80%–96%	[70]
*Luma apiculata*	MeOH (80%), 1:6, 1 h	TPC: 48–57 mg GAE/g FW. TFC: 0.55–0.98 mg QE/mL extract	DPPH (IC_50_): 17–21 mg/mL. ABTS: 9–16 TE/g FW. FRAP: 10–20 μM FeSO_4_/g FW. ORAC: 62.48 μmol TE/g DW	[71]
*Sambucus* spp.	Water, 1:5, r.t., 30 min	TPC: 3687–6831 mg GAE/kg FW	ABTS: 3.2–39.59 mM TE/kg FW	[72]
*Crataegus monogyna*	EtOH (45%), 1:10, r.t., 4 weeks	TPC: 0.8 mg GAE/mL	DPPH: 1147.67 mg AAE/L. FRAP: 531.42 mg AAE/L	[49]
*Prunus cerasus*	EtOH (42.39%, acidified 1% formic acid), 1:15, 40 °C, 75 min	TPC: 493.09 mg/L. TAC: 36.01 mg/L	ABTS: 59.61 mM Trolox/mL	[47]
*Ribes nigrum*	Aqueous SO_2_ (1000–1200 ppm), 1:19, 35 °C, 60 h	TPC: 89.4 mg CA/g FS DW. TAC: 15.8 mg C3G/g FS DW		[73]
*Vaccinium arctostaphylos*	MeOH (80%), 8:15	TPC: 11,291.4 ng/g FW		[74]
*Hippophae rhamnoides*	Soxhlet extraction: EtOH, 1:30, 8 h. Maceration: EtOH, 1:10, r.t., ovn.	TPC. Soxhlet: 4.9 mg GAE/g DW. Maceration: 2.3 mg GAE/g DW	DPPH: Soxhlet: 21.37 mg TE/g DW. Maceration: 14.28 mg TE/g DW. ABTS: Soxhlet: 8.33 mg TE/g DW. Maceration: 2.13 mg TE/g DW	[75]
*Myrtus communis*	EtOH (60, 70, 80, and 90%), 13:25, r.t., 40 days		DPPH: 65%–87.5%	[46]
*Vaccinium* spp.	EtOH (80%), 1:10, 24 h	TPC: 382 mg GAE/L extract. TAC: 160 mg/L extract	FRAP: 3.4 mM Fe^2+^ equivalents	[76]
*Vaccinium myrtillus*	EtOH (91.83%), 1.22, 18 °C, 23.5 days (for max. anthocyanin content) or 28 days (for max. phenolic content)	TPC: 3709.51 mg GAE/L extract. TAC: 2810.6 mg C3G/L extract	DPPH: 3689.38 mg AAE/L extract	[20]
*Dovyalis hebecarpa*	Acetone (20%, acidified 2% formic acid), 1:120, 20 min	TPC: 195 mg GAE/100 g pulp FW, 555 mg GAE/100 g skin FW. TAC: 69 mg CGE/100 g pulp FW, 284 mg CGE/100 g skin FW	ABTS: 5.8 μmol TE/g pulp FW, 20.8 μmol TE/g skin FW. FRAP: 10.3 μmol TE/g pulp FW, 29.7 μmol TE/g skin FW. ORAC: 50.1 μmol TE/g pulp FW, 135 μmol TE/g skin FW	[77]
*Rubus ellipticus* and *Rubus niveus*	MeOH (80%, acidified 1 N HCl), 2:5, 60 °C, 1 h	TPC: 2.56–3.28 mg GAE/g FW (*R. ellipticus*), 3.21 mg GAE/g FW (*R. niveus*). TAC: 0.01–0.28 mg/100 g FW (*R. ellipticus*), 5.63 mg/100 g FW (*R. niveus*). TFC: 4.58–4.71 mg QE/g FW (*R. ellipticus*), 4.91 mg QE/g FW (*R. niveus*)	DPPH: 26.36–27.72 mM AAE/100 g FW (*R. ellipticus*), 27.84 mM AAE/100 g FW (*R. niveus*). ABTS: 3.34–4.58 mM AAE/100 g FW (*R. ellipticus*), 2.97 mM AAE/100 g FW (*R. niveus*). FRAP: 2.19–3.43 mM AAE/100 g FW (*R. ellipticus*), 2.06 mM AAE/100 g FW (*R. niveus*)	[78]
*Vaccinium myrtillus*	Soxhlet extractions MeOH, EtOH, acetone and water, successively, 24 h	TPC: 116.67–182.33 μg CE/mg DW. TAC: 10.52–16.87 mg C3G/L extract. TFC: 23.94–37.49 μg CE/mg DW	DPPH: 13.59–25.40 μg/mL extract. FRAP: 53.73%–92.74% (using EtOH 89.70%)	[79]
*Rubus ellipticus*	MeOH (80%, acidified or not), 1:5, r.t., 30 min	TPC: 550–690 mg GAE/100 g FW. TFC: 179–276.6 mg CE/100 g FW	DPPH: 359.2–502.2 mg CE/100 g FW. ABTS: 619.6–704.9 mg BHAE/100 g FW. FRAP: 695.7–956.7 mg AAE/100 g FW	[80]
*Euterpe oleracea*	Acetone (50%), 7:4000, r.t., 1 h	TPC: 13.9 mg GAE/100 g FW.	ORAC: 997 μmol TE/g	[81]
*Morus alba*	EtOH (70%), 1:2, r.t., 4 h	TPC: 2235–2570 μg GAE/g DW. TAC: 1229–2057 μg/g DW	DPPH: 60%–80%	[82]
*Aristotelia chilensis*	MeOH (acidified 0.1% HCl)	TPC: 15,987 μmol TE/g extract	DPPH (IC_50_): 1.62 μg/mL. FRAP: 12,973.9 μmol CE/g extract (extract is 25 μg/mL). ORAC: 29,689.5 μmol TE/g extract (extract is 10 μg/mL)	[83]
*Vaccinium oxycoccos*	MeOH (acidified 0.1% HCl), 1:8, 15 min	TPC: 374.2 mg GAE/100 g FW. TAC: 77.1 mg C3G/100 g FW	DPPH: 68.8 μmol Trolox/g FW. ABTS: 16.4 μmol Trolox/g FW	[21]
*Rubus caucasicus*	Acetone:Water:Acetic acid (70:29.5:0.5), 1 h	TPC: 424 mg GAE/100 g FW. TAC: 168 mg C3G/100 g FW	DPPH: 37.4 μmol/g FW. FRAP: 56.30 μmol TE/g FW	[84]
*Vaccinium meridionale*	4 extractions with MeOH	TPC: 758.6 mg GAE/100 g FW. TAC: 329 mg C3G/100 g FW	ABTS: 45.5 μmol TE/g FW. FRAP: 87 μmol TE/g FW; 116 μmol Fe^2+^/g FW	[85]
*Vaccinium corymbosum* var. Bluecrop	Acetone (50%), 2:25 (peel), 6:25 (flesh)	TPC: 296.9 mg GAE/100 g flesh DW, 4142.3 mg GAE/100 g peel DW. TAC: 255.8 mg C3G/100 g flesh DW, 4750.4 mg C3G/100 g peel DW	ORAC: 287.5 μmol TE/g flesh DW, 958.9 μmol TE/g peel DW	[86]
*Aronia melanocarpa*	Absolute MeOH (acidified 0.3% HCl)	TPC: 1713 mg GAE/100 g FW. TAC: 277.13 mg C3G/100 g FW	ABTS: 171.7 μmol TE/g FW. FRAP: 206.2 μmol Fe^2+^/g FW. ORAC: 41.7 μmol TE/g FW	[87]
*Rubus idaeus*	MeOH:Water:Acetic acid (75:30:5)	TPC: 3.72 mg GAE/g FW. TAC: 11.95 mg C3G/100 g FW	ABTS: 2.12 mg AAE/g FW	[88]
*Morus alba*	4 extractions, EtOH (50%), 12 h each	TPC: 690.83 mg GAE/g FW. TAC: 272 mg C3G/g FW	DPPH: 698.57 mg TE/g DW. FRAP: 120.02 mg TE/g DW	[89]
*Aronia melanocarpa* and *Vaccinium corymbosum*	3 extractions, EtOH (70%), 1:10, 70 °C, 3 h each. 14.2% (*Aronia melanocarpa*), 8.7% (*Vaccinium corymbosum*)	TPC. 110 mg GAE/g (*Aronia melanocarpa*), 27.4 mg GAE/g (*Vaccinium corymbosum*)	DPPH inhibition at concentration of 10, 50, and 500 μg/mL were: 31.1%, 37% and 72.7% (*Aronia melanocarpa*), 29.4%, 29.6% and 40.6% (*V. corymbosum*), respectively. ABTS inhibition at concentration of 10, 50 and 500 μg/mL were: 4.6%, 10.3% and 46.3% (*Aronia melanocarpa*), 2.3%, 4.2% and 8.6% (*V. corymbosum*), respectively	[90]
*Fragaria x ananassa* var. Camarosa	Absolute EtOH or Acetic acid (0.2%), 1:20, 60 °C, ovn.	TPC (100 μg fruit extract): 207.4 mg GAE/g FW (EtOH), 224 mg GAE/g FW (Acetic acid)	DPPH (IC_50_): 39.01 mg/mL (EtOH), 29.86 mg/mL (Acetic acid). FRAP (IC_50_): 24.16 μg (EtOH), 57.11 μg (Acetic acid)	[91]
*Synsepalum dulcificu*	2 extractions absolute MeOH, 2:5, 60 °C, 30 min each	TPC: 1448.3 mg GAE/100 g flesh FW, 306.7 mg GAE/100 g seeds FW. TFC: 9.9 mg QE/100 g flesh FW, 3.8 mg QE/100 g seeds FW	DPPH: 96.3% (flesh). ABTS: 32.5% (flesh). FRAP: 22.9 mmol/100 g flesh extract	[92]
*Sambucus nigra*	6 different solvents: (A) Pure water; (B) 70% ethanol; (C) Pure methanol; (D) 70% Acetone; (E) Acidified methanol; (F) Infusion, 1:20, r.t., 5 days. Best efficiency: (E) (602 mg extract/g fruit DW)	TPC: 8974 mg GAE/100 g extract DW (A). TAC: HPLC (1326 mg C3G/100 g DW extract), pH-differential method (1066.6 mg C3G/100 g DW extract) (B)	DPPH (IC_50_): 117 μg/mL (D), 123 μg/mL (A). ABTS: 1.96 mM (D), 1.87 mM (A)	[93]
ULTRASOUND ASSISTED EXTRACTION (UAE)				
*Fragaria* spp.	MeOH (acidulated 0.20% HCl), 1:2, 20 °C, 10 min. Yield: 83%–99%	TAC: 63.25 μg/g		[13]
*Rubus fruticosus*	EtOH (64%, acidulated 0.01% HCl), 2:5, 35 kHz, 60 W, 25 °C and 40 °C, 15 or 30 min. Yield: 9.44% FW, 6.34% DW (40 °C, 30 min)	TPC: 2658 g GAE/100 g DW (40 °C, 15 min). TAC: 1.38 g C3G/100 g DW (40 °C, 30 min)	DPPH: 96 μg/mL (25 °C, 30 min). FRAP: around 190 μmol Fe^2+^/L at all conditions	[51]
*Myrciaria cauliflora*	EtOH (46%), 1:20, 25 kHz, 150 W, 30 °C, 60 min	TPC: 92.8 mg GAE/g DW. TAC: 4.9 mg C3G/g DW		[50]
*Aronia melanocarpa*	EtOH (50%), 1:20, 30.8 kHz, 100 W, 40 °C, 15 min. Yield: 84%	TPC: 1000 mg GAE/L extract (ratio 1:10), 600 mg GAE/L extract (ratio 1:20)	DPPH (IC_50_): 250 mg GAE/L extract	[1]
*Lonicera caerulea*	EtOH (80%, acidulated 0.5% formic acid), 1:25, 40 kHz, 100 W, 35 °C, 20 min	TPC: 107.93–527.50 mg GAE/100 g FW. TAC: 22.73 mg C3G/g DW, 99–329 mg C3G/100 g FW		[94]
*Rubus idaeus*	150 mL fruit puree without added solvent, 20 kHz, 400 W, 35 °C, 10 min	TPC: 1529 mg GAE/L. TAC: 317 mg C3G/L	DPPH: 7260 μmol/L	[16]
*Rubus strigosus* and *Vaccinium corymbosum*	Water, 1:1, 24 kHz, 400 W, 25 °C, 20 min	TPC *: 460 μg GAE/mL (*Rubus strigosus*), 500 μg GAE/mL (*Vaccinium corymbosum*). TAC *: 75 mg C3G/L (*Rubus strigosus*), 750 mg C3G/L (*Vaccinium corymbosum*)	DPPH *: 525 μmol TE/L (*Rubus strigosus*), 440 μmol TE/L (*Vaccinium corymbosum*)	[17]
*Rubus fruticosus, Morus nigra, V. myrtillus* and *Prunus spinosa*	MeOH (acidified 0.1% HCl), 1:4, 59 kHz, 25 °C, 60 min	TAC: 457.6, 301.9, 3888.1 and 476 mg C3G/L fruit extract for *Rubus fruticosus, Morus nigra, V. myrtillus* and *Prunus spinosa*, respectively	DPPH: 6.4, 1.6, 8.3 and 8.4 μmol TE/100 g FS for *Rubus fruticosus, Morus nigra, V. myrtillus* and *Prunus spinosa*, respectively	[18]
*Lonicera caerulea*	MeOH (acidified 0.1% HCl), 1:10, 90 min	TPC: 470–798 mg GAE/g DW. TAC: 401–457 mg C3G/L extract	ORAC: 52–68 μmol TE/g FW	[52]
*Crataegus monogyna*	EtOH (45%), 1:10, 30 min	TPC: 0.032 mg GAE/mL	DPPH: 56.73 mg AAE/L extract. FRAP: 105.25 mg AAE/L extract	[49]
*Aronia melanocarpa*	Water or EtOH (25% or 50%), 1:40, 30.8 kHz, 50 or 100 W, 45 °C, 240 min	TPC: >70 mg GAE/g DW. TAC: >13 mg CGE/g DW	DPPH: >450 μmol TE/g DW	[10]
*Prunus cerasus*	EtOH (40%), 1:15, 37 kHz, 40 °C, 40 min	TPC: 493.84 mg/L. TAC: 38.20 mg/L	ABTS: 105.87 mM Trolox/mL	[47]
*Hippophae rhamnoides*	Absolute EtOH, 1:10, 30 °C, 60 min	TPC: 3.8 mg GAE/g pulp DW, 4.4 mg GAE/g fruit DW	ORAC: 7.07 mg/g pulp DW, 16.72 mg/g fruit DW. ABTS: 4.86 mg/g pulp DW, 6.13 mg/g fruit DW	[75]
*Rubus* spp., *Vaccinium* spp., *Fragaria x ananassa* and *Aronia melanocarpa*	EtOH:Water:HCl (70:29:1), 1:10, 30 °C, 2 h	TPC *: 800, 700, 700 and 600 mg GAE/g DW for *Rubus* spp., *Vaccinium* spp., *Fragaria x ananassa* and *Aronia melanocarpa*, respectively. TAC *: 520, 610, 210 and 520 mg C3G/g DW for *Rubus* spp., *Vaccinium* spp., *Fragaria x ananassa* and *Aronia melanocarpa*, respectively	DPPH *: 5400, 3750, 4250 and 5500 μmol TE/g extract weight for *Rubus* spp., *Vaccinium* spp., *Fragaria x ananassa* and *Aronia melanocarpa*, respectively. ORAC *: 9000, 6750, 6900 and 4500 μmol TE/g extract weight for *Rubus* spp., *Vaccinium* spp., *Fragaria x ananassa* and *Aronia melanocarpa*, respectively	[48]
*Ribes nigrum*	EtOH (70%), 1:10, 100 kHz, 23–25 °C, 30 min	TPC: 3136.6 mg GAE/100 g DW. TAC: 182.4 mg cyanidin-3-rutinoside/100 g DW	DPPH: 94.7%	[24]
*Rubus coreanus*	EtOH, 40 kHz, 250 W, 54 °C, 37 min. Yield: 22.78%		DPPH: 80.94 μmol TE/g DW	[40]
MICROWAVE ASSISTED EXTRACTION (MAE)				
*Hippophae rhamnoides*	400 g press cake without added solvents (57% moisture content), 2.45 GHz, 1 W/g, 400 W, 15 min. Yield: 3% DW	TPC: 1147 mg GAE/g DW	DPPH (IC_50_): 0.71 g extract/L, 4.78 mmol GAE/L	[2]
*Lycium barbarum*	MeOH (25%–50%), 1:20, 0.38 W/g, 100 °C, 10 min	TPC: 9.2 mg GAE/g DW	ABTS: 7.6 mg AAE/g DW	[54]
*Hippophae rhamnoides*	4 g berries without added solvent (72% moisture content), 5 cycles of 1000 W (5 s), cooling system 20–25 °C between cycles		DPPH: 90%	[67]
*Vaccinium myrtillus, Vaccinium vitis-idaea, Vaccinium oxycoccos, Fragaria x ananassa, Ribes nigrum, Ribes rubrum*	3 extractions EtOH (70%), 1:2, 180 W, 3 min	TPC: 10.33–43.43 mg TAE/100 g FS	ABTS: 0.57–1.89 µM AAE/100 g FS	[95]
*Hippophae rhamnoides*	Absolute EtOH, 1:10, 150 W, 60 °C, 20 min	TPC: 9.3 mg GAE/g DW	DPPH: 28.40 mg TE/g DW. ABTS: 18.81 mg TE/g DW	[75]
PRESSURIZED LIQUID EXTRACTION (PLE)				
*Vaccinium myrtillus*	Absolute EtOH, EtOH (50%), Acidified water, EtOH (50%, acidified water), Acetone, 0.5–40 MPa, 25–180 °C, 15 min. Yield: 4.2% FS (Absolute EtOH), 8% FS (Acidified water)	TPC: 102 mg GAE/g DW, 87.1 mg GAE/g FW (absolute EtOH)	DPPH: 1867 μmol TE/g DW. ABTS: 103 μmol TE/g DW (absolute EtOH)	[3]
*Sambucus nigra*	EtOH (80%), 60 bar, 100 °C, 10 min	HPLC: 0.5288 g TAC/100 g, 0.2518 g C3G/100 g, 0.2018 g TFC/100 g	DPPH: 67.69%	[62]
SUPERCRITICAL FLUID EXTRACTION-CARBON DIOXIDE (SFE-CO_2_)				
*Hippophae rhamnoides*	SFE-CO_2_ with entrainer EtOH (30%), 345 bar, 44 °C, 80 min. Recovery of 90.82% tocopherol, 67.12% carotene		DPPH (IC_50_): 18.85 mg/mL	[43]
*Euterpe oleracea*	SFE-CO_2_ (900 kg/m^3^) 490 bar, 70 °C, 30 min. Yield: 45% DW	TPC: 5457–7565 mg GAE/100 g sample. (900 kg/m^3^, 350 bar, 70 °C). TAC: 96.1–137.5 mg/100 g sample. (700 kg/m^3^ 220 bar, 50 °C)		[55]
*Rubus idaeus*	SFE-CO_2_ (2 L/min) 45 MPa, 60 °C, 120 min. Yield: 14.61%. Residues re-extracted with MeOH:EtOH (50:50), 10.3 MPa, 30–110 °C, 5–25 min. Yield: 15% (hexane fraction), 25% (methanol fraction)	TPC: 26.31–38.95 mg GAE/g DW (MeOH), 5.37–10.15 mg GAE/g DW (Hexane)	ABTS: 308–561 μmol TE/g (MeOH), 48.5–122.7 μmol TE/g (Hexane). ORAC: 936.2 μmol TE/g (EtOH), 151.07 μmol TE/g (SFE-CO_2_)	[96]
*Vaccinium myrtillus*	CO_2_:Water:EtOH (90:5:5), 20 MPa, 40 °C, 1.4 × 10^−4^ kg/s. Yield: 1.96%	TPC: 134 mg GAE/g FW. TAC: 1071 mg/100 g FW	DPPH: 1658 μmol TE/g FW. ABTS: 199 μmol TE/g FW	[3]
*Vaccinium myrtillus*	SFE-CO_2_, EtOH (10%) first 30 min, then 2 SubC extractions with less EtOH each, 25 MPa, 45 °C	TPC: 72.18 mg GAE/g DW. TAC: 0.62 mg C3G/g DW	DPPH (IC_50_): 102.66 μg DW (SubC-CO_2_). ABTS (IC_50_): 8.49 μg DW (SubC-CO_2_). FRAP (IC_50_): 10.30 μg DW (SubC-CO_2_)	[56]
*Hippophae rhamnoides*	SFE-CO_2_, 46 MPa, 333 K, 6–7 h. Yield: 158.84 g/kg DW	424.1 mg total tocopherol/kg DW		[97]
Raspberry, blueberry and cranberry (species not specified)	SFE-CO_2_, 80–300 bar, 60 °C, 2.5 L CO_2_/h, 2 h. Yield: 5.20% (raspberry, 200 bar), 3.89% (cranberry, 250 bar), 1.4% (blueberry, 200 bar)	TPC (mg GAE/100 g pomace): 76.8 (raspberry, 80 bar), 29.5 (blueberry, 80 bar), 84 (cranberry, 250 bar)	DPPH (μg DPPH scavenged/g GAE): 89.5 (raspberry, 300 bar), 81.5 (blueberry, 250 bar), 109.9 (cranberry, 250 bar). ABTS (μg Trolox/g GAE): 21.79 (raspberry, 80 bar), 25.9 (blueberry, 200 bar), 5.35 (cranberry, 80 bar)	[98]
*Crataegus monogyna*	SFE-CO_2_, 5 L/min, 310 bar, 60 °C, 20 min	TPC: 0.303 mg GAE/mL extract	DPPH: 66.23 mg AAE/L extract. FRAP: 182.13 mg AAE/L extract	[49]
*Rubus glaucus*	SFE-CO_2_:EtOH (80:20), 140 bar, 32 °C, 65 min. Yield improved up to 59.3%	TAC: 85.4 mg C3G/kg FW		[99]
PULSED ELECTRIC FIELDS (PEF)				
*Vaccinium myrtillus*	Juice: 3 kV/cm. 55.5% yield. Cake press extract: 5 kV/cm. Treatment time: 1–23 μs	TPC: 109.1 mg GAE/100 mL juice, 1782.6 mg GAE/100 g FW berry press cake. TAC: 50.23 mg C3G/100 mL juice, 1699 mg C3G/100 g FW berry press cake	FRAP: 5–6.7 μmol TE/mL juice, 40–72 μmol TE/g FW berry press cake	[100]
*Rubus strigosus* and *Vaccinium corymbosum*	4 L puree + water, 1:1, 25 kV, 300 W, 66 μs	TPC *: 460 μg GAE/mL (*Rubus strigosus*), 490 μg GAE/mL (*Vaccinium corymbosum*). TAC *: 150 mg C3G/L (*Rubus strigosus*), 725 mg C3G/L (*Vaccinium corymbosum*)	DPPH *: 510 μmol TE/L (*Rubus strigosus*), 440 μmol TE/L (*Vaccinium corymbosum*)	[17]

* Given values are estimations from the graphics in the original papers. BHAE: BHA equivalents. CA: Chlorogenic Acid. CE: Catechin Equivalents. DW: Dry Weight. FS: Frozen Sample. HPLC: High Performance Liquid Chromatography. Max.: maximum. ovn.: overnight. QE: Quercitin Equivalents. r.t.: room temperature. SubC-CO_2_: Subcritical carbon dioxide extraction. TAE: Tannic Acid Equivalents. TFC: Total Flavonoid Content.

**Table 5:** Antioxidant content and capacity of *Vaccinium myrtillus* extracts obtained with different methods.

Extraction Method	Conditions	TPC, TAC ^1^	DPPH, ABTS ^2^, FRAP ^3^	References
Solvent extraction	Water, 80–100 °C, 4–15 min	1153 mg GAE/L extract	-	[66]
	EtOH, 28 days	3709.51 mg GAE/L extract	3689.38 mg AAE/L extract	[20]
Ultrasound assisted	MeOH acidified 0.1%, 59 kHz, 60 min	^1^ 3888.1 mg C3G/L extract	8.3 μmol TE/100 g FS	[18]
Microwave assisted	EtOH, 180 W, 3 min	43.43 mg TAE/100 g FS	^2^ 1.89 μM AAE/100 g FS	[95]
Pressure assisted	PLE, EtOH	102 mg GAE/g DW	1867 μmol TE/g DW	[3]
	Sub/Supercritical CO_2_	72.18 mg GAE/g DW	IC_50_: 102.66 μg DW	[56]
	Sub/Supercritical CO_2_	134 mg GAE/g FW	1658 μmol TE/g FW	[3]
Pulsed electric fields	Berry press cake, 5 kV/cm	1782.6 mg GAE/100 g FW	^3^ 40–72 μmol TE/g FW	[100]

^1^ TAC, ^2^ ABTS, ^3^ FRAP.

## Appendix A

**Table A1:** Botanical and common name of red fruits named through the review.

Botanical Name	Common Name
*Aristotelia chilensis* (Molina) Stuntz	Maqui
*Aronia melanocarpa* (Michx.) Elliott	Chokeberry
*Dovyalis hebecarpa* (Gardner) Warb.	Ceylon gooseberry
*Euterpe oleracea* Mart.	Açaí
*Fragaria* spp.	Strawberry
*Hippophae rhamnoides* L.	Seabuckthorn
*Lonicera caerulea* L.	Honeyberry
*Luma apiculata* (DC.) Burret	Chilean myrtle
*Lycium barbarum* L.	Goji berry
*Morus alba* L.	White mulberry
*Morus nigra* L.	Mulberry
*Myrciaria cauliflora* (Mart.) O. Berg	Jabuticaba
*Prunus cerasus* L.	Cherries
*Prunus spinosa* L.	Blackthorns
*Ribes nigrum* L.	Blackcurrant
*Ribes rubrum* L.	Redcurrant
*Rubus caucasicus* Focke	Caucasian raspberry
*Rubus coreanus* Miq.	Korean black raspberry
*Rubus ellipticus* Sm.	Golden Himalayan raspberry
*Rubus fruticosus* L.	Blackberry
*Rubus glaucus* Benth.	Andean blackberry
*Rubus idaeus* L.	Raspberry
*Rubus niveus* Thunb.	Ceylon raspberry
*Sambucus Nigra* L.	Elderberry
*Smilax aspera* L.	Rough bindweed
*Synsepalum dulcificum* (Schumach.) Daniell	Miracle-fruit
*Vaccinium arctostaphylos* L.	Caucasian whortleberry
*Vaccinium corymbosum* L.	Blueberry
*Vaccinium macrocarpon* Aiton	Cranberry
*Vaccinium meridionale* Sw.	Andean blueberry
*Vaccinium myrtillus* L.	Bilberry
*Vaccinium oxycoccos* L.	Small cranberry
*Vaccinium vitis-idaea* L.	Cowberry

## Appendix B

**Table A2:** Abbreviations used.

Abbreviation	Meaning
AAE	Ascorbic Acid Equivalents
ABTS	2,2-Azinobis-(3-ethylbenzothiazolin-6-sulfonic acid)
BHAE	BHA equivalents
C3G	Cyanidin-3-glucoside equivalents
CA	Chlorogenic Acid
CE	Catechin Equivalents
Cy	Cyanidin
DAD	Diode-Array-Detector
Dp	Delphinidin
DPPH	2,2-Diphenyl-1-picrylhydrazyl
DW	Dry Weight
FRAP	Ferric Reducing Antioxidant Power
FS	Frozen Sample
FW	Fresh Weight
GAE	Gallic Acid Equivalents
GRAS	Generally Recognized As Safe
HAT	Hydrogen Atom Transfer
HPLC	High Performance Liquid Chromatography
IC_50_	Concentration at which 50% of radicals are scavenged
MAE	Microwave-Assisted Extraction
max.	Maximum
MHG	Microwave Hydrodiffusion and Gravity
MS	Mass Spectroscopy
Mv	Malvidin
ORAC	Oxygen Radical Absorbance Capacity
ovn.	Overnight
PEF	Pulsed Electric Field
Pg	Pelargonidin
PLE	Pressurized Liquid Extraction
Pn	Peonidin
Pt	Petunidin
QE	Quercitin Equivalents
r.t.	Room temperature
RSM	Response Surface Methodology
SE	Solvent Extraction
SET	Single Electron Transfer
SFE-CO_2_	Supercritical carbon dioxide extraction
SubC-CO_2_	Subcritical carbon dioxide extraction
TAC	Total Anthocyanin Content
TAE	Tannic Acid Equivalents
TE	Trolox Equivalents
TFC	Total Flavonoid Content
TPC	Total Phenolic Content
UAE	Ultrasound Assisted Extraction
